# Metagenomic Sequencing of Bronchoalveolar Lavage Samples from Feedlot Cattle Mortalities Associated with Bovine Respiratory Disease

**DOI:** 10.1128/genomeA.01045-17

**Published:** 2017-10-05

**Authors:** Devin B. Holman, Cassidy L. Klima, Brenda J. Ralston, Yan D. Niu, Kim Stanford, Trevor W. Alexander, Tim A. McAllister

**Affiliations:** a Lacombe Research and Development Centre, Agriculture and Agri-Food Canada, Lacombe, Alberta, Canada; b Lethbridge Research and Development Centre, Agriculture and Agri-Food Canada, Lethbridge, Alberta, Canada; c Alberta Agriculture and Forestry, Airdrie, Alberta, Canada; d Alberta Agriculture and Forestry, Lethbridge Research Centre, Lethbridge, Alberta, Canada

## Abstract

Bovine respiratory disease (BRD) is a significant and costly illness in feedlot cattle. Metagenomic analysis was performed on bronchoalveolar lavage samples obtained from 18 feedlot cattle that died of BRD.

## GENOME ANNOUNCEMENT

Bovine respiratory disease (BRD) is the most frequent cause of morbidity and mortality in feedlot cattle, resulting in significant economic losses in the North American beef cattle industry (1). It is a multifactorial disease associated with stress and several different bacterial and viral agents (2). Bronchoalveolar lavage (BAL) samples were collected postmortem from 18 feedlot cattle that died of bovine respiratory disease in Alberta, Canada. The BAL samples were obtained from animals in either conventional or natural (antimicrobial-free) feedlots in 2016. The use of BAL samples rather than deep nasopharyngeal swabs allows for the characterization of the microbiome in the lungs where BRD often manifests as bronchopneumonia (3). It may also identify novel agents associated with BRD mortalities.

The BAL samples were filtered to remove bovine cells, and microbial cells were then pelleted via centrifugation. Metagenomic DNA was extracted from each BAL sample as described by Beukers et al. (4), with the exception that mutanolysin was added to aid bacterial lysis. Metagenomic libraries were generated using the NEBNext Ultra II DNA library prep kit (New England Biolabs, Whitby, ON, Canada) and sequenced on a HiSeq 2500 (Illumina, Inc., San Diego, CA, USA) instrument using the HiSeq Rapid SBS kit v2 (500 cycles; Illumina, Inc.). Reads were trimmed using Trimmomatic (5) v0.36, and bovine reads were subsequently removed using Bowtie2 v2.2.9 (6), SAMtools v1.4.1 (7), and BEDtools v2.25.0 (8). Taxonomy was assigned to the host-filtered reads using Kaiju v1.5.0 (9) and the NCBI nonredundant protein database. Megahit v1.1.1 (10) was used to assemble the metagenomic reads, and BLASTn was then used to identify contigs matching the comprehensive antibiotic resistance database [CARD] (11) for resistance determinants.

The most abundant bacterial species among the BAL samples were *Mannheimia haemolytica* (18.8%), *Bacteroides pyogenes* (14.3%), *Pseudomonas taetrolens* (5.7%), *Anaplasma phagocytophilum* (5.0%), *Clostridium perfringens* (3.8%), *Mycoplasma bovis* (3.2%), *Psychrobacter* sp. SHUES1 (2.7%), *Histophilus somni* (2.5%), and *Streptococcus pneumoniae* (2.1%). The most frequently detected antibiotic resistance genes were *aadA*, *floR*, *tet*(40), *tet*(H), *tet*(Q), *tet*(W), and *sul2*. The metagenomes obtained in this study represent the first characterization of the lower respiratory tract in BRD-related mortalities.

### Accession number(s).

The metagenomic sequences have been deposited in the NCBI Sequence Read Archive under accession numbers SRR5877067 to SRR5877084.
